# Electronic Vapor Cigarette Battery Explosion Causing Shotgun-like Superficial Wounds and Contusion

**DOI:** 10.5811/westjem.2016.1.29410

**Published:** 2016-03-02

**Authors:** Siri Shastry, Mark I. Langdorf

**Affiliations:** University of California Irvine Medical Center, Department of Emergency Medicine, Orange, California

## INTRODUCTION

Electronic vapor cigarettes (E-cigarettes) were created in 2003 as an alternative to traditional tobacco cigarettes. E-cigarettes have been available in the United States since 2006.^1^ The typical E-cigarette consists of a cartridge that contains liquid, an atomizer that heats the liquid (i.e. acts as a vaporizer), as well as a battery. The liquid contained within the cartridge contains nicotine, propylene glycol and/or glycerol as well as flavorings. The consumer uses an E-cigarette through either pushing a button or inhalation, which triggers heating and therefore aerosolizes the liquid within the cartridge, emulating cigarette “smoke.” The newest E-cigarettes are larger than nicotine cigarettes and employ stronger, rechargeable batteries as a power source.^2^,^3^

These new devices allow for custom modifications instituted by the user, including various options for cartridges and for the heating temperature of the atomizer. This creates the opportunity for mismatched components, leading to overheating. The current literature is limited on both the long-term health impact of E-cigarette use as well as the mechanical safety of these devices.^2^,^3^ We present the second documented case of burn injury due to explosion of an E-cigarette, and the first instance of the explosion occurring during usage of the device.^4^

### Case Report

A 26-year-old man presented to the emergency department (ED) via ambulance with purported burns to his left shoulder and chest. Thirty minutes prior, the patient had been smoking an E-cigarette at his house. The patient is a paid tester for an E-cigarette company, and the cigarette he was using was an experimental model. The cigarette was a customizable, large device powered by a lithium-ion battery. The patient denied misuse of the cigarette at the time of injury. The E-cigarette exploded while the patient was using it and the battery subsequently disintegrated into diffuse shrapnel. This material, as well as the ejected battery, struck the patient diffusely on the upper abdomen, left shoulder and chest. The patient further noted that across the room was a three-inch hole in the drywall about five feet above the floor, and 8–10 feet from him. It was unclear which part of the device became the projectile that damaged the wall.

There was no reported loss of consciousness, and the patient denied headache and head or neck injury. The patient did not fall to the ground. There was no resulting fire or significant smoke concerning for inhalation injury.

The patient has a history of smoking tobacco and marijuana, but does not use other drugs or alcohol. The patient had no other past medical/surgical history. Review of systems was otherwise negative.

On physical exam, the patient’s vital signs were the following: temperature 36.9C, HR 50bpm (sinus bradycardia), RR 16/minute, BP 131/85 and O_2_ saturation of 98% on room air. The patient had soot in his nares and skin of his neck, but otherwise had no evidence of facial burns. He had no soot in the oropharynx, and nasal/facial hair was not singed. He had broad peppering of the skin on the left chest, abdomen and anterior/medial left arm with a small area of second-degree burn versus foreign body penetration in the left upper quadrant of the abdomen and the anterior chest superior to the left nipple. The patient also had soot over the left hand, with which he had been holding the device, with small penetrating foreign bodies in the palmar surface of the thumb. The patient had superficial skin pain over these sites.

Heart and lung sounds were normal. His abdomen was soft with mild to moderate tenderness over the contused areas but no rebound or guarding. His extremities had full range of motion, no deformity, and no apparent penetration of foreign bodies into any joint. An extended-focused assessment of sonography of trauma (E-FAST) revealed appropriate lung sliding and was negative for free fluid in the peritoneum and pericardium.

**Figure 1:**
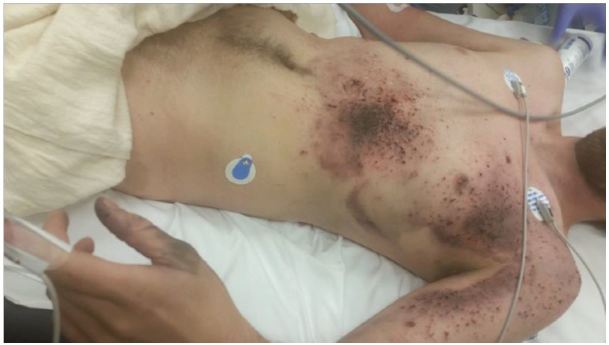
Shrapnel-like superficial skin penetration and contusion from exploding lithium-ion battery from an electronic vapor cigarette.

**Figure 2:**
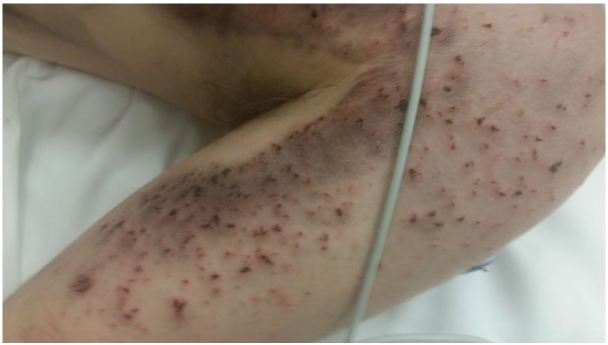
Closeup of patient’s left upper arm after explosion of the lithium-ion battery from an electronic vapor cigarette, with superficial schrapnel-like wounds.

**Figure 3:**
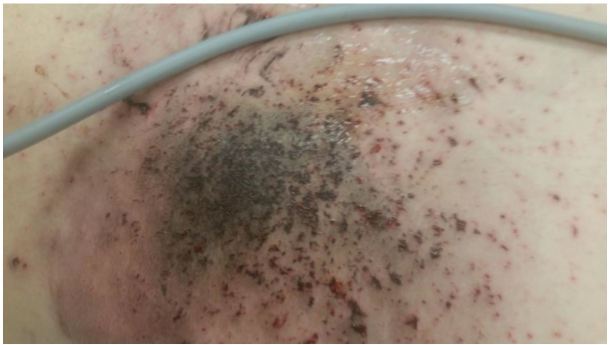
Closeup of patient’s left upper quadrant contusion/schrapnel-like injury after explosion of electronic vapor cigarette lithium-ion battery.

**Figure 4:**
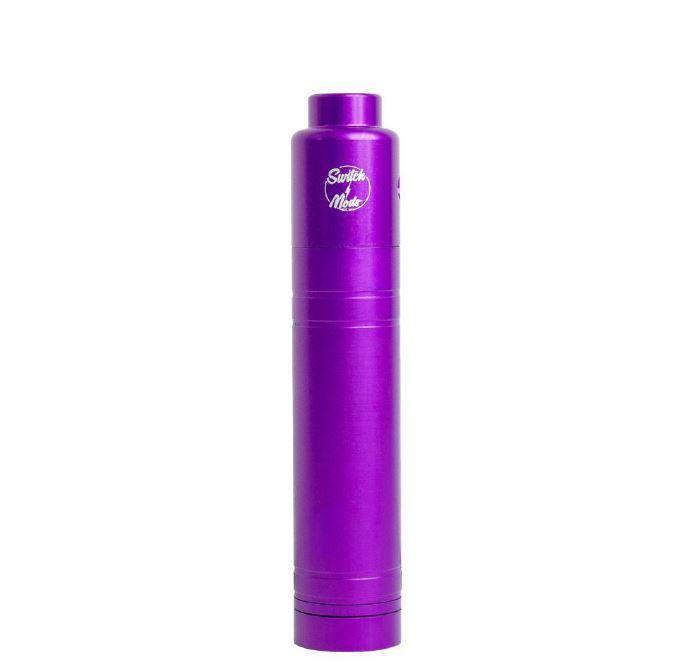
Electronic vapor cigarette cartridge device that exploded in use by patient (2.4cm in diameter and 10cm in length).

**Figure 5:**
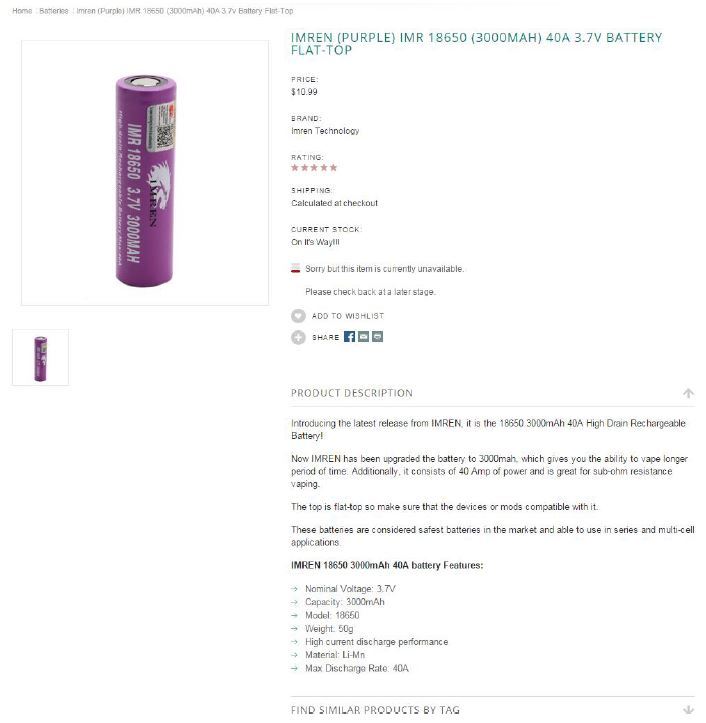
Lithium-ion battery contained in electronic vapor cigarette device that exploded with patient use.

Representative photographs of the patient’s injury are presented in Figures 1, 2, and 3, and the device and battery in Figures 4 and 5.

The patient tested positive for tetra-hydra-cannabinol (THC) but toxicology screen was otherwise negative. All other lab results were unremarkable. Initial chest radiograph showed no significant abnormalities. Despite the shrapnel-like appearance of the skin injury, there were no radio-opaque foreign bodies. Computed tomography (CT) of the abdomen/pelvis showed soft tissue contusion anterior-superiorly in the midline subcutaneous fat but no evidence of acute intra-abdominal injury. Pulmonary CT angiography showed multiple soft tissue contusions and skin thickening anteriorly, but no acute intrathoracic injury. There was no pneumothorax, pulmonary contusion, solid organ injury or peritoneal fluid.

The patient did not have debridement of foreign bodies from the skin while in the ED, as there did not appear to be any discrete removable fragments. He was discharged home from the ED after approximately six hours of observation with recommendations for daily wound care and follow up with the burn clinic in two weeks.

At the patient’s follow-up appointment, he endorsed continued use of the E-cigarette. The patient expressed concerns about scarring but denied pain or discomfort. He was back at work without difficulty. Physical exam showed scattered tattoo residue from the explosion on the chest and left upper arm, but good healing of lesions. There was a midline, tender 2.5×3.5cm abdominal wall nodule with some ecchymosis, which may be a retained foreign body. The patient was scheduled for a return visit in 4–6 weeks.

## DISCUSSION

This patient presented with extensive soft tissue lesions on the chest, abdomen and upper arm due to explosion of a lithium-ion battery while using an E-cigarette. At this time, E-cigarettes are sold with minimal oversight from any regulatory body, and there are limited data on both long-term and immediate health risks.^2^,^3^ A majority of E-cigarette users believe them to be a safer alternative to nicotine cigarettes, and view them as a path to smoking cessation or reduction.^5^ While there has been speculation regarding long-term effects, including carcinogenesis of some heated liquid components, there has been minimal consideration of mechanical risks.

Lithium-ion batteries, commonly used to power these devices, are a known explosion hazard. Lithium-ion batteries in laptops and cellphones have been implicated in incidents similar to the presented case.^6^ Lithium-ion batteries may overheat during charging or when exposed to the liquid in the E-cigarette cartridge, leading to “thermal runaway,” an unregulated increase in internal battery temperature. Exposure of the lithium-ion batteries to elevated temperatures can lead to explosion.^7^ Various engineering strategies have been proposed to mitigate the risk, but without regulatory oversight or standardized manufacturing, there is great variation in materials and processes that produce E-cigarettes.^3^,^6^ Our patient’s device advertises on its website, “venting holes to reduce battery heat by 60%,” and “maximum copper contact to battery surface area.” The device retails for $120 and the battery for $10.

These risks are largely uncharacterized in the medical and even lay media due to recent advent of this technology. However, there have been at least five media reports in the past three months of overheating battery explosions in the U.S., causing shrapnel injuries, facial burns, penetrating injuries to the leg, facial and cervical spine fractures, tongue lacerations and contusions, broken teeth and an amputated index finger. In California, there are media reports of three personal injury lawsuits filed by November 2015.

One paper recommended against using E-cigarettes as an alternative to tobacco smoking in patients with chronic obstructive lung disease on home oxygen due to risk of fire.^8^ However, there were no other citations on the topic of the explosive or burn injury dangers of E-cigarettes in a PubMed search.

Accordingly, physicians treating E-cigarette users should advise patients of these risks. From a public health/safety standpoint, the E-cigarette industry should take these risks into consideration during manufacturing and advertising, so as to protect and inform consumers.
